# Is the EU shirking responsibility for its deforestation footprint in tropical countries? Power, material, and epistemic inequalities in the EU’s global environmental governance

**DOI:** 10.1007/s11625-023-01302-7

**Published:** 2023-02-18

**Authors:** Eric Mensah Kumeh, Sabaheta Ramcilovic-Suominen

**Affiliations:** 1 Bioeconomy and Environment Unit, Natural Resources Institute Finland, Itäinen Pitkäkatu 4 A, 20520 Turku, Finland; 2 Leverhulme Centre for Nature Recovery, Environmental Change Institute, School of Geography and Environment, University of Oxford, Oxford, UK

**Keywords:** Socio-ecological conflicts, Decolonial environmental justice, Transformations, Just globe

## Abstract

This paper critically examines the European Union’s (EU) role in tropical deforestation and the bloc’s actions to mitigate it. We focus on two EU policy communications aimed at the challenge: stepping up EU action to protect and restore the world's forests and the EU updated bioeconomy strategy. In addition, we refer to the European Green Deal, which articulates the bloc’s overarching vision for sustainability and transformations. We find that by casting deforestation as a production problem and a governance challenge on the supply side, these policies deflect attention from some of the key drivers of tropical deforestation—the EU’s overconsumption of deforestation-related commodities and asymmetric market and trade power relations. The diversion allows the EU unfettered access to agro-commodities and biofuels, which are important inputs to the EU’s green transition and bio-based economy. Upholding a ‘sustainability image’ within the EU, an overly business-as-usual approach has taken precedence over transformative policies, enabling multinational corporations to run an ecocide treadmill, rapidly obliterating tropical forests. Whereas the EU's plan to nurture a bioeconomy and promote responsible agro-commodities production in the global South are relevant, the bloc is evasive in setting firm targets and policy measures to overcome the inequalities that spring from and enable its overconsumption of deforestation-related commodities. Drawing on degrowth and decolonial theories, we problematise the EU’s anti-deforestation policies and highlight alternative ideas that could lead to more just, equitable and effective measures for confronting the tropical deforestation conundrum.

## Introduction

Since 2017, the One Planet Summit has convened annually to reaffirm world leaders’ commitments to address the unfolding ecological crisis. In January 2021, many world leaders joined the Summit virtually because of the COVID-19 pandemic. The 2021 Summit focused on biodiversity and the need to protect ecosystems in the interest of human health. Speaking on the topic, multiple world leaders emphasized the link between ecosystems, rural livelihood, and the need for more investment in nature-based solutions. One voice that stood out in the keynote addresses was Angela Merkel, then Chancellor of Germany. Speaking on ecosystems degradation and biodiversity loss, Merkel passionately appealed to her peers, admonishing:

‘Natural habitats are being destroyed every day. We risk losing around a quarter of most plant and animal species. These drastic losses have a serious impact on life and quality of life, including for us humans. And so, we must step up our efforts to protect biodiversity and natural habitats—*not some time or other, but now, and not somehow or other, but monumentally*. *If we do not, the consequences will soon be irreversible*’ (Merkel 2021, p. 1, *Author emphasis*).

Within half a year from the summit, in July 2021, many countries in Western Europe were blighted by floods that their sophisticated disaster management systems struggled to manage effectively. Two hundred and thirty people died from the flood, with Germany hit hardest—184 people died there. While the world was reeling from this climate shock, Hurricane Ida unleashed its fury on the USA, tragically snatching at least 60 more lives and displacing several others. While these two disasters dominated the media in July, it is worth noting that extreme floods tragically took even more lives in the global South within the same month (Davis 2021): 192 in Mumbai and Maharashtra (India); 113 in Nuristan Province, Afghanistan, and 99 in Henan Province, China, to name a few. July of 2021 also saw wildfires ravage multiple countries, devasting multiple homes, livelihoods, and biodiversity. There is emphatic evidence that a growing likelihood of climate disasters stems partly from decades of excessive fossil fuel consumption and its associated emissions from centuries of human domination over nature (IPCC 2018, 2021). A fundamental approach to reducing emissions for the EU is fostering a bio-based economy or bioeconomy (European Commission 2012; European Commission 2018).

The bioeconomy is central in debates within the EU on decarbonization and greening of the global economy. While definitions of the bioeconomy tend to vary across countries and sectors due to asymmetries in technology, infrastructure and natural resources endowments, its central tenet is the replacement of fossil fuels with biological materials in all sectors of the economy (European Commission 2012, European Commission 2018; Wesseler and Braun 2017). As research and investments in the bioeconomy take root, multiple crevices have emerged in the approach, with three being particularly dominant. First, some scholars argue that the bioeconomy is rooted in ecological modernization logic in ways that place humans above and beyond nature (Ramcilovic-Suominen 2022; Ramcilovic-Suominen and Pülzl 2018; Vivien et al. 2019). The second, and related crevice, focuses on the neoliberal and profiting from nature mentality within which the bioeconomy has evolved (Kleinschmit et al. 2017; Kröger and Raitio 2017). Still, a third argument is that the bioeconomy perpetuates existing material inequalities, and epistemic and ontological injustices by using the ‘global North’ visions of sustainability and development to reproduce the exploitation of resources such as land, forests, and labour from the global South (Backhouse 2021; Bastos Lima 2021). Building on these arguments, some scholars are concerned that the bioeconomy would deepen socio-ecological conflicts, deforestation, and inequalities, not only but predominantly in the global South (Backhouse et al. 2021). Within these assessments, the bioeconomy can be understood as an approach designed to snooze the alarm on facing the unfolding ecological crisis and divert attention from raging debates and calls, especially by youths, for radical and systemic transformations (Ramcilovic-Suominen et al. 2022). These concerns unsettle the EU’s framing of the bioeconomy as a sustainable development strategy. They disconcert the EU’s (hereafter: bloc) self-representation as the beacon of global sustainability, including its commitments to addressing its contributions to deforestation and in the global South (European Commission 2019b, p. 20). Is the EU shirking responsibility for its deforestation footprint in tropical countries?

Multiple studies point out how the EU’s focus on competitiveness and economic growth leads the bloc to outsource its enormous ecological footprints to the global South (Hickel et al. 2021; McMichael 2017). As a high-income bloc, the EU has a per capita material throughput of 27 tonnes per annum, which is more than 13 times the average person in a low-income country (International Resource Panel (IRP) 2019). Maintaining such high material throughput in the bloc and other high-income regions relies on the disproportionate exploitation of land, labour and raw materials from the global South, drawing upon unequal material and power relations that evolved from the global North’s colonisation of the former (Alonso-Fernández and Regueiro-Ferreira 2022; Hickel et al. 2021). Besides, decades of the global North’s meddling in the global South, including through its structural adjustment programmes (SAPs) imposed on many global South countries in the 1980s, is documented to have deepened the former’s control over many countries in the region, carving them open for multinational companies to exploit their natural resources, peoples and environment further (McMichael 2017). To address these unequal power and economic relations and their impacts on human and more-than-human entities and beings (McGregor et al. 2020), some scholars emphasize the need for decolonial approaches that questions the global North’s ‘imperial mode of living’, which thrives off normalising the domination and control of low-income nations—their nature, people and culture—to reproduce production and distribution systems that enable overconsumption of resources mainly for economic growth over social and environmental sustainability (Brand and Wissen 2018, pp. 46–48). An emerging body of knowledge on decoloniality and degrowth (“Unequal relations and structures of oppression: past, present, and future”) calls for different ways of living as opposed to peripheralization and the creation of dichotomous socio-natures that divide to conquer and extirpate some human and more-than-human entities and beings, knowledge systems and ways of life (Abazeri 2022; Ramcilovic-Suominen 2022).

By situating the deforestation question within dependency, degrowth and decoloniality theories, this paper critically examines whether emerging EU policies seek to deliver meaningful changes to the bloc’s role in causing deforestation in the global South, particularly through its import of agro-commodities such as soy, palm oil, cocoa, coffee, and biomass, notably wood pellets and bioethanol. The paper pays attention to two of the bloc’s policy communications that speak to deforestation-causing commodities—i.e., ‘Stepping up EU Action to Protect and Restore the World’s Forests’ (hereafter: SAPReF) and the EU Updated Bioeconomy Strategy (UBS)—due to how tropical deforestation directly affects climate, biodiversity and peoples’ ways of living in global South (Hoang and Kanemoto 2021; Kumeh et al. 2022; Urzedo and Chatterjee 2021), and the policy window currently available within the bloc to explore solutions to tropical deforestation (European Commission 2021).

## Unequal relations and structures of oppression: past, present, and future

A common thread in crevices of the bioeconomy, as an approach for future-proofing society and nature, is how actors advance and contest the (re)production of seemingly natural imaginaries that entrench exploitative power and economic relations that underpin the ongoing socioecological crises. These imaginaries manifest in at least three reinforcing folds. First, bioeconomy proponents posit it as a tool to reproduce Europe’s competitiveness, which is likely to reinforce rather than address the global inequality crises. The second is a human–nature binary and hierarchy that conceitedly situates humans above other species. Third, an onto-epistemological orientation that largely perceives ‘scientific exploration’ as the primary and only way out of the climate emergency. Invariably, these imaginaries are reinforced by the global North’s cultural and material dominion in the global South through centuries of colonisation that obliterated other forms of knowledge and nurtured unequal material exchange relations prevalent in the global economy. Many postcolonial and decolonial and some degrowth scholars emphasize the need to resist such logics, pointing out the need to go beyond Eurocentric visions of human–nature relations and associated solutions and address the historically rooted and embedded causes, such as colonialism, violence, slavery, material plunder and ontological and epistemological dominance. Such discussions have been at the core of decoloniality, radical and indigenous environmental justice (McGregor et al. 2020; Whyte 2020), and degrowth studies (Abazeri 2022; Hickel 2021a, b), which we turn to further our analyses.

### Unequal exchange, power, and economic relations: from modernisation through dependency to degrowth

Multiple frameworks address the relationship between economic development and the environment, which are relevant for exploring inequality and the need for differentiated responsibilities in the deforestation question. Modernisation theory collocate trade and comparative advantage as inalienable to development, attributing the underdevelopment of “poor countries” to their inability to break from tradition and internalise modernity values that nurture development (Webster 1990). Dependency approaches, on the other hand, reject these assumptions, arguing that “poor countries” are poor because they have been colonised and their human and natural capacities and resources plundered for the development of the global North. Dependency approaches, therefore, emphasize that poor countries are not poor because of their internal circumstances but because the terms upon which they are integrated into the global economy enable the North to siphon the former’s resources to enrich themselves at the expense of the latter (Amin 1972). Recognising the asymmetry in resource flow between rich and poor countries in the global economy, some scholars position countries along a core-periphery world order, with Wallerstein (1974) nuancing a semi-periphery as a category between the dichotomous typologies, possessing attributes of both core and periphery. The literature indicates that economic inequality persists due to the global North’s desire to remain at the core of the global hierarchy while relegating the global South to the periphery—mainly as subordinates that are sources of cheap raw materials and labour required for production and further surplus capital accumulation in the North (Alonso-Fernández and Regueiro-Ferreira 2022; Hickel et al. 2021). Yet, acknowledging the strong relationship between per capita footprint and levels of economic development, some scholars use unequal ecological exchange theory to explain how the global North appropriates resources and economic surplus from the global South whilst externalizing the environmental burdens from economic production to the latter (Jorgenson 2006, p. 687). More recently, some studies argue that an excessive focus on exchange value over the use value of resources in the global economy with the imperative of relentless capital accumulation nurtures overconsumption, whereby many actors and countries in the North use resources that far exceed what it needs to remain within safe planetary boundaries (Stuart et al. 2020). Bunker (1988, p. 16) argues that understanding the relations that facilitate overconsumption, embedded in unequal ecological exchange, requires deconstructing the historical context within which ‘relations between world systems of exchange and the social, economic, and political organization’ have emerged between countries in the global North and South. Studies have attempted to approach this from material and onto-epistemological perspectives.

Starting with the material, Jason Hickel provides a pulsating historical analysis of how the North deprives many countries in the global South of their resources in *The Divide: Global Inequality from Conquest to Free Markets*. Hickel’s work reveals how the global North plundered the South of mineral resources to fuel the Industrial Revolution while simultaneously dwelling on slavery and imports of cheap nourishment from the South to free labour in the North for industrialisation. Moreover, his account illuminates how economic and military power accumulated from industrialisation in the North enables it to influence and enforce international institutions that indebt, impoverish the global South, and deepen inequalities in the global economy. Using the World Bank, the International Monetary Fund (IMF) and the World Trade Organisation (WTO) as cases, Hickel demonstrates how the global North influences trade rules and peddles questionable financial schemes to undermine the ability of many countries in the South to build buoyant economies. One example in the literature is how the IMF, through its Structural Adjustments Programs (SAPs), institutionalises a debt burden on many countries in the global South (Hickel 2018).

Through SAPs, the North is argued to have carved open economies of the South for exploitation, imposing stringent prerequisites to aiding them during the 1980s debt crisis. These included ‘commercial and financial liberalization, deregulation of goods, capital and labour markets, privatization, the elimination of consumer and producer subsidies, cuts in social spending’ (Bourguignon 2015, pp. 109–110). Some studies indicate that since the beginning of SAPs, the global South have transferred about USD 13 trillion to the North as debt service payments, which could have been invested in industrialisation and social services to reduce poverty (Hickel 2018, p. 124). Besides, the South loses about USD 161 billion yearly in unequal exchange due to the North’s ability to employ its market power to undervalue materials, labour, and other resources it imports from the South (Hickel 2018). In a recent work covering 1990 and 2015, Dorninger et al. (2021) found that apart from China and India, all other countries were net exporters of embodied resources to the global North. Meanwhile, the value added per ton of raw material and unit of embodied labour were 11 and 28 times more in the global North than for the lowest income countries in the global South. These not only allow the global North to appropriate and overconsume resources from the South and externalise their environmental impacts, but it also facilitates its ability to generate economic surpluses to bolster its dominance in the global hierarchy (Dorninger et al. 2021).

The excessive focus on economic growth is proven to be at the heart of the surging ecological and inequality crises, which are increasingly used as the basis to call for degrowth economies in the Global North (Hickel 2021a). Degrowth literature is on the ascendency in global development scholarship, primarily for the argument that green growth aspirations are misguided for at least two reasons. First, multiple studies indicate that absolute decoupling cannot be achieved globally under the logic of continuous economic growth (Giampietro 2019; Parrique et al. 2019). Second, scholars and activists caution that amid the climate emergency, there is simply no time left; therefore, we can no longer snooze the alarm for drastic actions, hoping that technology may come to our rescue (Hickel 2019, 2021a, b). Degrowth scholars and activists advocate for envisioning and planning new economic systems that work to reduce our material throughput, which reduces exploitation and expropriation of resources and labour, and which instead prioritise community, human wellbeing, and ecological care (Akbulut 2021; Hickel 2021a, b). They advocate for ways of living beyond the ‘imperial mode of living’, which thrives on the overconsumption (Brand and Wissen 2018; Eversberg 2020), and emphasize the need for anti-establishment, pioneering, revolutionary options to reject the overconsumption logic embedded in economic growth, including the destruction, erasure, and pain it inflicts on the environment and people of the global South (Dunlap 2022).

Hickel (2021a, b, p. 1107) argues that economic growth is a propaganda term because of how well it succeeds in masking the destruction of socio-natures caused by its intrinsic logic of accelerating material throughput. Whereas many world leaders use economic growth to signal an increase in the gross domestic product (GDP), a growing body of literature demonstrates its illusions, highlighting the need to unveil the multiple imaginaries that are masked with this signification (Bebbington et al. 2018; Hickel et al. 2021; Schaffartzik et al. 2019; Torvikey 2021). Jason Hickel puts this well when he writes that growth is not about abstract gross domestic product (GDP); instead, it is an increase in material throughput, consumption and accumulation, and thus, ‘the ongoing process of elite accumulation, the commodification of commons, and the appropriation of human labour and natural resources—a process that is quite often colonial in character’ (Hickel 2021a, b, p. 1107). In this way, degrowth is about breaking the hegemony of growth and unsettling/challenging or reframing power relations that have historically ostracised other forms of knowing and being to appropriate resources for growth. Degrowth calls for ‘disaccumulation, decommodification, and decolonization’ (Hickel 2021a, b), creating spaces for actors and states to focus on their own wellbeing instead of strong-arming them to cave into serving as sites of exploitation, violence and destruction (Ramcilovic-Suominen 2022). Responding to this call means creating space for solutions that are radically different from the green growth approaches currently available (Kothari et al. 2014). Solutions that transcend the peripheralization of regions of the world in other to exploit them and destroy their environment (Barlow et al. 2016; Costello et al. 2013; Dunlap 2021; Pendrill et al. 2019; Urzedo and Chatterjee 2021).

### Onto-epistemological deprecation and the need for just and decolonial turn

Another factor that enables the North to reinforce its dominance in the global economy is its ability to downgrade the values that contradict the logic of ecological modernisation. Imperial Europe’s project of the Colonial Era, wrapped in the illusion of “civilising savage cultures”, thrived on subjugating the desires of European colonies by casting their culture as “backwards”, “savage”, and their ways of knowing as ‘inferior’ to the western ways of knowing and being. Nkrumah captures this subjugation mechanism well, writing:

‘Even if we were no longer, on the evidence of the shape of our skulls, regarded as the missing link, unblessed with the arts of good government, material, and spiritual progress, we were still regarded as representing the infancy of mankind. Our highly sophisticated culture was said to be simple and paralyzed by inertia, and *we had to be encumbered with tutelage*.’ (Nkrumah 1964, p. 82, *Author emphasis*).

This onto-epistemological deprecation, bridled with the political and cultural cleansing, birthed and continues to nurture exploitative relations between the North and the South (Ndlovu-Gatsheni 2015; Quijano 2007). Yet, amid the rubbles and ruins of centuries of European plunder, in the South lie different ways of relating and living in harmony with nature and humanity between and across generations. Unearthing these are at the heart of decolonial discourses.

Decoloniality evolved as a critique of development that racialize, short-circuit, and erase cultures, knowledge, race, and gender for economic growth. It does not seek to dismiss achievements of modernity summarily. Instead, it seeks to reorient humanity to rediscover, re-learn and restore knowledge and ways of being that have been or are being ostracised, forgotten, and interred by racial capitalism and the colonial matrix of power that reinforces it (Gram-Hanssen et al. 2021; Ndlovu-Gatsheni 2015). Decoloniality scholars distinguish between colonialism and coloniality in trying to question the seeming naturalness and universality of Western knowledge and culture to restore racialised socio-natures. Colonialism, they argued, is the process of colonisation that ended with the declaration of political independence in colonies, while coloniality characterises the extension of colonialism through institutional, economic and other matrices of power built by colonialism (Quijano 2007).

In *Consciencism: Philosophy and Ideology for De-colonization and Development, with particular reference to the African Revolution*, Nkrumah (1964, p. 68) avers that man is primarily regarded in indigenous Africa as a ‘spiritual being endowed originally with a certain dignity, integrity and value’. A man’s relation with others, he argues, is ‘the initial equality of all, and the responsibility of many for one.’ In Southern Africa, this relation manifests in *Ubuntu*, a moral ethic and way of life, which holds that ‘I am because we are’, also rendered ‘a person is a person through others’ (Gade 2011). For Nkrumah, this ethic—where the shared welfare of the people is supreme, where a person essentially becomes more human through affirmed positive relations with others—renders it impractical for the emergence of Marxian classes, characterised by disproportionate economic and political power between people, where ‘certain classes are crushed, lacerated and ground down by the encumbrance of exploitation’. Construed so, a raging struggle continues among scholars on Ubuntu's emancipatory and transformative potential. On the one hand, scholars contend the potency of *Ubuntu*-based policy to deliver radical transformation, associating its rise with black elitism, which coincided with the end of colonialism (Matolino and Kwindingwi 2013). Even so, such scholars and their critiques recognise *Ubuntu’s* embodiment of African humanism, forged on the anvil of care, harmony and solidarity, can provide for a variety of normative and transformative relations that radically contrast prevailing Western relations that have crippled our earth systems (Gram-Hanssen et al. 2021).

Concerning multispecies justice and relational ontology, it is not far-reaching to extend *Ubuntu* to non-human attributes because of indigenous African’s deep ties to non-human species. Indeed, many studies point out that *Ubuntu* describes and illustrates the relations between human and non-human elements of the cosmos (Mawere 2014). Many traditional African societies experience and relate to the earth as a ‘mother’, viewing it not only as a provider but also as the home of the Ancestor, which requires of them the responsibilities of solidarity and care (Chibvongodze 2016)—a view considered fundamentally different from the individualistic-capitalistic orientation of the global North (Terblanché-Greeff 2019). Abandoning dualism between the human and non-human elements of the cosmos is not limited to *Ubuntu*. It is embodied in the lifestyles of multiple indigenous peoples across South and North America and wider (McGregor et al. 2020; Whyte 2020). Various scholars and activists draw on the environmental justice struggles in the region to demonstrate how indigenous peoples of the region strive to resist, overturn and transcend the primary focus on overconsumption in the North and drives epistemicide and ecocide in the region (Dunlap 2021; de Santos 2014). Concurrently, they point out how communities in the region are wrestling to re-exist according to their own ways of knowing and being attuned to maintaining harmony in the cosmos, a harmony radically imperilled by racial capitalism. A defining task, such activists, aver, is *Buen vivir*, a transformative model of good living that displaces the capitalist mode of production and its logic of viewing nature as a resource reserve to satisfy human desires and (re)construct social relations that determine and are affected by means of production (Álvarez and Coolsaet 2020; de Santos 2014). These growing calls from erstwhile largely ostracised global South actors impose a moral responsibility on humanity that is distinct from the logic underlying the North’s conceptions of responsibility in addressing tropical deforestation in at least two ways.

First, the morality described is irreducibly relational, whereby self-actualisation is colloidally bound to one’s ability to relate with others positively. This means that any action that focuses on building an individual interest, for example, by exploiting others (human and other than human species) or being indifferent to communal interest to gain a competitive advantage, contravenes collective harmony and is, therefore, disingenuous. Second, a positive relationship with others within the indigenous ethic is explicitly communal, prioritising ways of living that safeguard harmony, solidarity, and care. This contrasts with the North’s capitalist morality, which rationalises relations according to individuality, safeguarded under notions of private property, participation, and informed consent. The difference is that whereas this morality, under the flagship of democracy, prioritises the needs and demands of the majority, the indigenous ethic makes achieving harmony a normative aspiration that should guide the majority’s desires. Viewed through this lens, the extent to which an actor assumes or shirks responsibility within the tropical deforestation question amid the surging ecological crises can be delineated, and alternative visions articulated. For example, one can begin to gauge responsibility in the tropical deforestation question by simply asking whether an actor’s contributions to the problem and tackling it contributes to or impedes the broader global community’s aspirations to combat climate change and inequalities, which imperil the existence of both human and the other than human species.

## Tropical deforestation and bioeconomy: EU’s shares and responsibilities

The EU and, more broadly, the global North contribute significantly more to global emissions than less wealthy countries. In 2017, “high-income countries had a material footprint per capita^1^ of 27 tons, which is 60% more than a person from an upper-middle-income country and a humungous 1350% more than individuals in low-income countries”^2^ (International Resource Panel (IRP) 2019, p. 8). The uneven footprint is not confined to 2017; rather, it is redolent of economic production and consumption patterns that underpin the widening economic inequalities between the global North and South (Givens 2018; Hickel et al. 2021; Schaffartzik et al. 2019). Besides, much of the North’s production thrives on the net appropriation of resources and the destruction of ecosystems, including forests in the Global South (Dorninger et al. 2021; Pendrill et al. 2019; Winkler et al. 2021). Such net appropriation has colonial roots with many studies pointing out how the North’s desire for resources for surplus capital accumulation caused deforestation in many countries across Africa, Asia, and Latin America from the sixteenth century. These occurred through the direct exploitation of timber and mineral resources in many colonies while simultaneously transforming local self-sustaining agricultural systems in the colonies to produce forest-depleting commodities predominantly for the global North (Lambin and Geist 2003; Urzedo and Chatterjee 2021). The latter relied on clearing large swaths of forests and discipling cheap labour to establish plantations and infrastructure for export commodities such as tobacco, cocoa, oil palm, tea, and rubber, to name a few, in, among others, Brazil, Indonesia, India, Sri Lanka, Vietnam and Madagascar (Byerlee 2014; Daniel et al. 2019). Both pathways combined with the North’s control over the global economy compels many countries in the South to exploit and transfer a disproportionate amount of their land, raw materials, and labor resources to service the former’s imperial mode of living (Hickel 2018). While recognizing the essence and impacts of these historical structures, it is essential to illustrate how they manifest in current trade relations that underlie the tropical deforestation question. In delineating the EU’s shares in tropical deforestation and its related emissions, we focus on deforestation-risk commodities and biomass, including soy, palm oil, cocoa, wood pellets and bioethanol question based on our interests in debates on tropical deforestation and its relevance for the bloc’s ongoing search for options to mitigate its contributions to tropical deforestation (European Commission 2021).

### Agro-commodities

The European Commission (EC) recognises that the EU is a ‘net importer of commodities such as tropical fruits, coffee, tea, cocoa, soy products and palm oil, used as food and feed’ (European Commission 2018, p. 47). Studies that attempt to establish the scale of the EU’s deforestation footprint use trade information to extrapolate deforestation embodied in imports to EU countries (Pendrill et al. 2019). Wedeux and Schulmeister-Oldenhove (2021) estimate that the EU contributed to 16% (203,000 hectares) of deforestation embodied in international trade in 2017, a figure second to China (24%, 304,000), albeit the latter being more than thrice the population of the former. Between 2005 and 2017, the EU’s imports deforested about 3.5 million ha of tropical forests, i.e., 286,000 ha per annum (Pacheco et al. 2021). Soy, palm oil, beef, wood products, cocoa and coffee imports accounted for 84% of the EU’s deforestation in the tropics and sub-tropics, i.e., soy (89,100 ha: 31.12%), palm oil (69,200 ha, 24.21%), beef (28,700 ha, 9.69%), wood (22,500 ha, 7.86%), cocoa (17,600 ha, 6.15%), and coffee (14,500 ha, 5.10%) (Pacheco et al. 2021; Wedeux and Schulmeister-Oldenhove 2021).

On a country level, at the time of writing, Germany, Europe’s largest economy, was also the EU’s biggest contributor to tropical deforestation, an estimated 43,700 ha annually (Wedeux and Schulmeister-Oldenhove 2021). Italy (35,800 ha/year), Spain (32,900 ha/year), Netherlands (29,600 ha/year) and France (26,300 ha/year) complete the top five EU tropical deforesters from 2005 to 2017. Meanwhile, the UK also imported 360,000 ha of deforestation over the same period, i.e., 30,000 ha per annum. Overall, the EU’s imported deforestation between 2005 and 2017 emitted 1807 million tonnes of carbon dioxide, about 40% of the bloc’s annual emissions (Wedeux and Schulmeister-Oldenhove 2021). As with the area of exported deforestation, Germany and Italy led emissions from imported deforestation over the period, with 21.6 million tCO_2_ and 17.4 million tCO_2_ per annum, respectively. The United Kingdom (16.6 million tCO_2_), Netherlands (16.4 million tCO_2_) and Spain (16.3 million tCO_2_) were among the five EU countries with the most annual emissions embedded in their tropical commodities import.

Most of the EU’s tropical deforestation footprint is argued to occur in 24 countries in the global South, across Latin America, Africa, Asia and Oceania, referred to as ‘deforestation fronts’ (Pacheco et al. 2021, p. 8). On these fronts, Brazil, Argentina, Bolivia and Paraguay lose the most forests due to the EU's soy and beef imports (Goldman et al. 2020; Pacheco et al. 2021). Similarly, Indonesia and Malaysia are the main hubs of deforestation to service the EU's overconsumption of palm oil (Goldman et al. 2020). Across Africa, Cote d’Ivoire, Ghana and increasingly Nigeria and Cameroun are the leading countries deforested by EU cocoa imports (Fountain and Huetz-Adams 2020; Wessel and Quist-Wessel 2015). The region produces more than 70% of the world's cocoa; Table 1 shows the leading importers of cocoa produced globally (1975 and 2020).

**Table 1:** Cocoa (beans, butter, and powder) imported into the EU (1000 tonnes) 1975–2020

Country, region	Year									
	1975	1980	1985	1990	1995	2000	2005	2010	2015	2020
The Netherlands	152 (9.8)	176 (11.3)	278 (13)	368 (14.1)	458 (15.6)	591 (15.2)	801 (16.3)	861 (16.9)	536 (10.2)	1285 (18.4)
Germany	210 (23.4)	234 (14.9)	307 (14.3)	377 (14.4)	392 (13.4)	377 (9.7)	416 (8.4)	578 (11.3)	668 (12.7)	735 (10.5)
France	80 (5.1)	104 (6.6)	109 (5.1)	155 (5.9)	222 (7.6)	309 (8)	345 (7)	308 (6)	322 (6.1)	392 (5.6)
UK	111 (7.1)	118 (7.6)	139 (6.5)	196 (7.5)	208 (7.1)	216 (5.6)	226 (4.6)	165 (3.2)	133 (2.5)	191 (2.7)
EU-27	694 (51.6)	764 (56.4)	999 (53.2)	1260 (55.6)	1596 (61.5)	2013 (57.5)	2411 (53.5)	2615 (54.5)	2624 (52.4)	3688 (55.6)
World—(EU-27 + UK)	757 (48.4)	684(43.6)	1002 (46.8)	1165 (44.4)	1128 (38.5)	1650 (42.5)	2292 (46.5)	2323 (45.5)	2507 (47.6)	3101 (44.4)
World	1563	1568	2141	2622	2933	3880	4930	5104	5265	6980

Source: FAOSTAT. Figures in the brackets depict a percentage of the global total cocoa import

Over time, countries in the South that produce agrocommodities’ share of economic returns from trade have diminished even as their total production has increased. Evidently, this stems from the disproportionate market power of multinational corporations underpinned by the global North’s control over commodity pricing. For example, coffee producers used to retain 20% of the total income from the coffee trade in the 1970s and 1980s; however, today, they retain less than 10% (Utrilla-Catalan et al. 2022). A similar trend is observable in the cocoa sector, where Fountain and Huetz-Adams (2020) note that although West Africa (Cote Ivoire, Ghana, Nigeria) produces over 70% of the world’s cocoa, actors in the region receive a meagre 5–7% of the value generated from cocoa while the bulk of the value is retained in the global North. The unequal distribution of value from agrocommodities exchange thrives from the systemic undervaluing of land, labour, and raw materials from the South by the North. Yet, many countries in the South are required to mobilize ever more funds to service their financial debts, some of whose principal has been paid many times over, to the Global North (Hickel 2018). Evidently, this debt trap deepens unequal economic and ecological exchange, with countries in the global South destined to expend ever more resources to produce more economic gains for the North at the expense of the South’s social, economic and environmental integrity.

### Biomass

Biomass is an essential part of the EU’s plan to achieve climate neutrality by 2050, and it accounted for nearly 60% of the bloc’s renewable energy use in 2016 (European Commission 2019a). During the period, the bloc imported 4% of its 140 Mtoe. However, the bloc’s biomass import is forecast to grow significantly if it is to meet its climate goals (European Commission 2019a; Proskurina et al. 2019). Wood pellets are an essential element of the EU’s biomass import and its connection to the deforestation question. The bloc is a net importer of pellets, with its net imports rising to 14 million m^3^ in 2015; this is more than a ninefold increase from 2009 reference levels (Camia et al. 2021). While tropical countries have traditionally played a less significant role in pellet export to the EU (the USA, Canada, and Russia are the top three), the bloc’s pellet imports are projected to shift towards Southeast Asia due to the abundance of primary forest feedstock and lower cost of production in the region (Proskurina 2018). And while this shift appears to be underway (Calderón et al. 2019), fallouts from Russia’s invasion of Ukraine, including EU leaders’ decision to reduce the blog’s dependence on Russian energy sources, could accelerate this shift further, with countries such as Vietnam, Malaysia and Thailand likely to fill any supply gaps.

Biofuels, liquid or gas fuels generated from biomass, e.g., biodiesels and bioethanol, are the other forms through which the EU imports deforestation. About 46% per cent of palm oil imported into the EU is converted to biofuels, requiring the use of about one million hectares of tropical soils (Flach et al. 2017). As noted earlier, the EU palm oil imports deforested 69,200 ha annually in the tropics between 2005 and 2017 (Wedeux and Schulmeister-Oldenhove 2021). While the EU plans to phase out its palm oil use as biofuel to mitigate deforestation are well-placed (Rifin et al. 2020), there are also concerns that the bloc’s simultaneous aspiration to meet at least 32% of its energy demands from renewable sources may impose new demands elsewhere, including on bioethanol markets (Follador et al. 2021). Invariably, the EU can produce most of the bioethanol it needs; the bloc’s bioethanol production was 8.5 billion liters in 2013, quadruple of the 2.1 billion liters the bloc produced in 2006. However, it currently operates at about 60% of its production capacity, with production having stymied since 2012 and is predicted to stagnate further due to its preference for cheaper imports from Brazil (Kapustová et al. 2020). Follador et al. (2021) estimate that the EU could import about 30% (1.13 billion liters) of Brazilian ethanol by 2030, which would require an additional 4.6 million hectares of agricultural land and thereby increase pressure on deforestation in the Amazon. Meanwhile, there is ample evidence of how the pursuit of cheap commodities from the Amazon by the North has historically destroyed biodiversity (Buchadas et al. 2022; Hoang and Kanemoto 2021) and continues to imperil the existence of indigenous communities (Ferrante and Fearnside 2020), including their deep ecological knowledge and history of living in harmony with nature (Sze et al. 2022).

The EU recognizes that its trade imports drive tropical deforestation. Evidently, the desire to address tropical deforestation is not lost in political discourses within the EU. On the contrary, multiple European leaders acknowledge the need to find solutions to the problem, averring that tackling deforestation is essential for peace, stability, and prosperity in the tropics. For the EU Commissioner on Environment, Oceans and Fisheries, Virginijus Sinkevičius, “deforestation is an emergency, and we are determined to act … As European Commissioner, I am committed to tackling this challenge with efficient and mandatory measures under our European Green Deal”. Sinkevičius made this statement at the end of public consultation in December 2020, which received nearly 1.2 million responses, urging the EU to tackle deforestation. A month later, Ursula von der Leyen noted:

‘Being a major economy and trading superpower comes with responsibilities. And it is our duty to ensure that our Single Market does not drive deforestation in local communities in other parts of the world. This is why, later this year, we will propose new legislation to minimise the risk of products linked to global deforestation being placed on the EU market. Europe is ready to lead the way, and I hope others will join us in that effort!’ (European Commission 2022, p. 2)

She made this statement during the 2021 One Earth Summit, mentioned in the “Introduction”. At the very least, these public proclamations indicate some form of desire by the EU to mitigate its tropical deforestation footprint. But do these political statements translate into transformative policies that halt the bloc’s contribution to tropical deforestation?

## The EU’s response to tropical deforestation: bypassing the critical drivers of deforestation—overconsumption and various forms of inequality

The perception that world leaders are not doing enough to address the surging climate and equality crises is increasingly recognized and accepted (Ramcilovic-Suominen 2022; Samper et al. 2021). A critical reading of the EU’s policies for addressing tropical deforestation caused by its import of tropical agro-commodities and biomass, such as the SAPReF and UBS policies, highlights the risk of reinforcing material and racial inequalities as well as tropical deforestation. Both policies are underpinned by neoliberal approaches that seem only to worsen deforestation in the tropics, casting doubts about their potency. We argue that the EU’s overconsumption of deforestation-related commodities and trade inequality, both fuel the production and trade in such commodities, remain largely unaddressed. Both policies portray “deforestation-risky” countries as underdeveloped with weak governance systems requiring external help. This framing masks the EU’s logics for offering such help, which has historically been to discipline the South for the continued supply of cheap labor and raw materials, and as an area that is deeply dependent on the bloc. The need to protect biodiversity and forests is similarly articulated through an archetypical colonial lens of enclosures and protection that largely ignores the local indigenous and non-indigenous local communities’ ways of life and their livelihoods. Thus, the logic of living as and with nature that underpins the indigenous ethic risk being sacrificed on the altars of better governance participation and consent (McGregor et al. 2020; Whyte 2020). We begin with an overview of these policies before discussing them further.

### A short overview of the two policies: *‘Stepping up the EU action to protect and restore the world’s forests’* and *‘A sustainable Bioeconomy for Europe’*

The European Green Deal (EGD) is the EU’s overarching policy for transitioning its economy and society in a way that can achieve economic growth within planetary boundaries. Focusing on energy, transport, infrastructure, agriculture, and the environment, the EGD is built on three main pillars: finance, just transition, and the EU’s role as a global leader in sustainable development politics. In the EGD, the EC notes that it will “take measures to promote imported products and value chains that do not involve deforestation and forest degradation” in line with its earlier communication (European Commission 2019c, p. 14). The earlier communication referenced is “*Stepping up EU Action to Protect and Restore the World’s Forests*, (SAPReF)”, published in July 2019.

In SAPReF, the EC acknowledges that “the world’s forests are in danger from deforestation and forest degradation, with a forest area of 1.3 million square kilometres lost between 1990 and 2016.” (European Commission 2019b, p. 1). Moreover, the Commission recognized the EU’s contributions to the problem and its moral obligation to act, observing that ‘deforestation is not somebody else’s problem’ (European Commission 2019b, p. 6). It observed that while multiple regulatory and non-regulatory measures were already in place to tackle the problem, they were inadequate. Thus, new approaches are required to ‘protect and grow the world’s forest cover to improve people’s health and livelihoods and ensure a healthy planet for our children and grandchildren’ (European Commission 2019b, p. 6). To achieve this, the EU seeks to pursue the following under SAPReF: (i) ensure sourcing and consumption of deforestation-free commodities, through legislation and various certification schemes, (ii) leverage development cooperation to support tropical countries to create protected areas, strengthen their forest policy, and undertake sustainable farming, (iii) improve international cooperation for forest restoration, (iv) encourage the private sector to invest in sustainable land-use, and (v) promote research and sharing of EU expertise on the circular economy, bioeconomy, and climate-smart agriculture with other countries.

The Update European Bioeconomy Strategy, ‘*A sustainable bioeconomy for Europe: strengthening the connection between economy, society and the environment*’ (hereafter: UBS), was promulgated in 2018. The UBS followed a review of the EU’s 2012 bioeconomy strategy, and it seeks to address five strategic objectives, namely (1) ensure food and nutrition security; (2) promote sustainable natural resource management; (3) reduce dependence on non-renewable, unsustainable resources from EU and oversees; (4) mitigate and adapt to climate change; (5) create jobs and strengthen European competitiveness. It proposes three key measures. First, boost investments in the bio-based sector. Second, deploy local bioeconomies rapidly across Europe; third, invest in research to understand the ecological boundaries of the bioeconomy. These measures are anchored on circularity and sustainability, which are the fundamental principles in the UBS (European Commission 2018, p. 50). The UBS aims at sustainable natural resource management and proposes the need for the EU to use bio-based materials, including those produced and refined within European biorefineries (European Commission 2018). However, as shown in “Tropical deforestation and bioeconomy: EU’s shares and responsibilities”, the EU continues to import biomass from other markets that have a comparative advantage (i.e., cheaper and more available natural resources and labour compared to Europe) in biomass production, including tropical countries (Mai-Moulin et al. 2019; Mandley et al. 2020; Proskurina 2018).

### A critical reading of the SAPReF and the UBS: what about addressing overconsumption and various forms of inequality?

The policy instruments advanced by SAPReF and the UBS certainly advance certain ways in managing tropical deforestation, as proposing supply measures are part of the solution. Yet, focusing primarily on supply ignores the EU’s overconsumption of tropical commodities and inequalities associated with the unequal exchange of agrocommodities. Without addressing these critical causes, tropical deforestation and pushing local livelihoods and ways of life to extinction may persist (Costello et al. 2013; Rosa et al. 2016; Stévart et al. 2019).

To be clear, both the SAPReF and the UBS recognise Europe’s role as a “net importer of commodities such as tropical fruits, coffee, tea, cocoa, soy products and palm oil, used as food and feed” (European Commission 2018, p. 47). One estimate found that tropical deforestation accounts for 15% of the average EU diet (Pendrill et al. 2019). Still, more is embodied in unequal exchange, characterised by the bloc’s use of its power and economic powers to importing of deforestation-inducing commodities cheaply, process, and export them for surplus capital accumulation (Schaffartzik et al. 2019). Given how this logic of accumulation continues to drive tropical deforestation, one would expect that the bloc articulates plans to reduce its reliance on tropical agro-commodities for economic growth. Evidently, both policies recognise the issue of consumption in contextualising the tropical deforestation conundrum. Yet, the bloc shows little ambition in properly confronting its consumption, relegating policy options primarily to monitoring and voluntary measures. For example, although SAPReF recognises the need to ‘reduce the EU consumption footprint on land’ (European Commission 2019c, p. 7), it sets no basis upon which progress can be measured. This is concerning given that the policy also recognises that the EU’s imports of agro-commodities could lead to the disappearance of moist forests in, for example, Cote d’Ivoire, the EU’s biggest supplier of cocoa, by 2024 (European Commission 2019b, p. 3). With no clarity on the scale to which the EU intends to reduce its deforestation footprint, SAPReF envisions awareness creation, certification, and eco-labelling as mechanisms to influence EU consumers to choose deforestation-free commodities. These strategies are not new, and while useful, studies have challenged their effectiveness in reducing tropical deforestation due to, among others, “leakages, poor transparency and difficulties in implementing traceability, selective adoption and their tendency to marginalise smallholders in the global South” (Lambin et al. 2018, p. 109).

According to the UBS, the EU intends to develop and deploy biorefineries to utilise multiple biobased materials efficiently. The strategy acknowledges that the “siting of biorefineries will depend heavily on the profit margins of bio-based products”, and among others, “availability of local and/or regional feedstock at competitive prices, suitable infrastructure including logistics and skilled personal”. While these indicate that the bloc’s focus remains strongly on competitiveness and economic growth, the UBS is silent on the EU’s role in biomass import. This occurs despite predictions that the bloc’s import of wood pellets and bioethanol from the tropics will increase in the future (Follador et al. 2021; Mai-Moulin et al. 2019; Proskurina 2018). Concerned about how the EU’s imports of biomass could accelerate deforestation in other parts of the world, the European Economic and Social Committee (EESC), critiquing the UBS, cautions that “respecting sustainability principles is essential for a ‘new’ bioeconomy, and natural resources have to be conserved to keep them productive”. “Consequently”, the EESC continues, “the bioeconomy must follow sustainability criteria to avoid distortions to the disadvantage of the environment, economy and society, the same rules shall apply for biomass from the European Union and from abroad” (European Economic and Social Committee (EESC) 2019, p. 38). However, this is not explicitly addressed in UBS, which insists on the centrality of biomass for decarbonising Europe without a reckoning of its impacts deepening inequality and environmental destruction in the South.

By shelving the EU’s tropical agro-commodities and biomass overconsumption question, the bloc avoids articulating a critical plan essential for instigating much-needed changes in the consumption habits of its citizens. Focusing predominantly on the supply-side policies shifts the burden of responsibility onto countries in the global South, the smallest beneficiary of agro-commodity trade (Hickel et al. 2021) while risking the sustenance of multiple smallholders in the South (Zhunusova et al. 2022). Besides, this approach mistakenly and unfairly reduces the tropical deforestation conundrum to a set of governance challenges and the need for capacity building and monitoring deforestation on the supply side. The bloc positions itself as a capable leader in regulating deforestation-risk countries’ operations by providing guidance, logistic and economic incentives. Specifically, the EC avers that the EU needs to, inter alia: “ensure deforestation is included in the national dialogue in tropical countries”, “help partner countries develop and implement frameworks for sustainable forest management”, “help partner countries implement sustainable forest-based value chains” and “share innovative EU practices with partner countries” (European Commission 2019b).

SAPReF indicates that deforestation would be tackled through legislation in supply countries, certification of products, and capacity building where European knowledge and expertise are transferred to supply countries. Meanwhile the effectiveness of these approaches has been questioned repeatedly in the literature (e.g., Büscher et al. 2012; Zhunusova et al. 2022). On the one hand, the EU’s portrayal of the deforestation question as a governance challenge eerily echoes Nkrumah’s observation that imperial Europe has the proclivity for casting countries in the global South as sites ‘unblessed with the arts of good government’ to create a fertile ground for interventions that consolidate Europe’s control over such countries (Nkrumah 1964, p. 82). On the other, it feeds the dependency relationship that the EU has historically cultivated with countries in the South and continues to reproduce in a bid to amplify its ability to benefit disproportionately from trade in the global economy. While such an aspiration might serve the individualist aspirations of the bloc, it does not bold well for building the collective harmony required to adequately tackle the searing socioecological crises. Alternatively, by calling for a Eurocentric approach that seeks to insulate the EU markets from deforestation-risk commodities through legislation, SAPReF risks creating leakages and thereby shifting trade in deforestation-risk commodities to other areas, including China and the USA, which are equally interested in accumulating surplus capital and reproducing dependency relations with countries in the South.

Second, and even more illustrative of the dependency conundrum, SAPReF and the UBS do not adequately acknowledge, and address inequalities embedded in the production, trade and consumption of products that cause tropical deforestation. As noted earlier, tropical deforestation is embedded within the global economic and trade structures where the global North has the privilege of exploiting the land, forests and labour from tropical countries (Alonso-Fernández and Regueiro-Ferreira 2022; Hickel et al. 2021). There is considerable evidence that inequality produces and exacerbates tropical deforestation (Ceddia 2019; Ferraro and Simorangkir 2020; Miyamoto 2020) because many countries in the global South need to exploit ever more for their forest resources to raise revenue to pay off their debts to the global North (Hickel 2018; McMichael 2017). SAPReF recognises that forests are essential to “reduce global income inequality” (European Commission 2019b, p. 2) and that deforestation is a source of ‘income inequality’ (European Commission 2019b, p. 3), which is a welcoming shift in the EU’s official narrative. But it does not propose direct measures to tackle the inequalities embedded in tropical agro-commodities trade. It merely proposes redirecting finance into sustainable land-use practices, and investing in better monitoring and understanding of the problem, for example, by establishing an “EU observatory on deforestation” and “improving coordination among research institutes” (European Commission 2019b, p. 16)—as if there was a doubt and lack of evidence about the scale of challenge. Meanwhile, the word inequality is largely omitted from the UBS, which also ignores the nature and scale of the EU’s biomass imports from the tropics (Lühmann 2021; Ramcilovic-Suominen 2022).

The poor attention to global inequality in the UBS coupled with a lack of clear strategies, measures, and targets on how to address the challenge in both policies is concerning, given that many of the proposed tools for addressing deforestation (e.g. legislation and certification) impose additional obligations on poor and marginalised actors (Santika et al. 2021; Waldman and Kerr 2014). Glasbergen (2018, p 243) highlights these concerns when he writes that ‘smallholders do not eat certificates’, drawing attention to the inequities and burdens coffee and oil palm certification schemes impose on smallholders in low income countries.

The need to address various forms of inequality in agro-commodities trade is dire due to its impacts on the living conditions of people in the global South (Fountain and Huetz-Adams 2020; McMichael 2017; Utrilla-Catalan et al. 2022). While the two policies attempt to accommodate some of the concerns, they reduce the debate on inequalities to the distribution of benefits from production and trade. Addressing inequality requires confronting the EU’s entitlement and ability to impose governance and legality standards on economically less privileged countries (Ramcilovic-Suominen and Mustalahti 2022). It requires shifting from the individualist logic that encourages and overlooks elitist representation mechanisms known to obscure or misrepresent forest communities' voices and their daily experiences and relations with their environment (Kumeh et al. 2022; Myers et al. 2020). It also requires discussing historic and present contributions and responsibilities for ecological degradation, climate change, unequal opportunities and terms exchange between different countries. These call for more systemic changes, shifting from entrenching the South as geography for cheap resources and its people as subordinate and subservient to the EU’s growth ambitions to more collaborative, less exploitative partnerships focused on nurturing local autonomy, and social and ecological harmony. Decolonial and degrowth approaches would seek to articulate alternatives that would enable tropical countries to take control of their production system and economic and food sovereignty, providing them with fairer outcomes while reducing exploitation, various forms of inequality, and violence.

## Rethinking the EU’s role in addressing tropical deforestation

Tropical deforestation, as observed today, is rooted in centuries of European imperialism, commodification and accumulation of forestlands, labour, and other resources and commodities in the tropics. The consistent signification of the global North’s worldviews and ways of life, including the idea of progress and development, where consumerism and GDP growth are fetishized, has created and expanded a wasteful economy with unsustainably high material throughput (Stuart et al. 2020). Reducing the overconsumption of tropical commodities in the EU requires deemphasizing the logic of economic growth, wealth accumulation and European domination and competitiveness, which underprop unequal economic and ecological exchange in the global economy. Breaking away from consumerism is fundamental for tackling over-reliance on tropical agrocommodities, its associated deforestation and inequalities. In what follows, we provide a few entry points for moving in this direction.

### Scaling down consumption of deforestation-causing commodities (in the EU)

The unfolding socioecological crises, including climate change, biodiversity loss and global inequalities, cannot be addressed without changing the mindsets, logics, rethinking of our roles and relationships with each other and other beings and with that our understanding of progress and development. Rethinking the capitalist global economy, where multinational companies enhance their economic and political power, across different scales and geographies, is central. Tropical deforestation persists partly due to a solid demand to consume and accumulate surplus capital from tropical agrocommodities and biofuel. Embracing self-imposed frugality and striving to reduce the proportion of deforestation commodities imported to Europe is not a matter of individual choice; however, as the economic system requires, promotes and portrays it as desirable. The problem is not individual but systemic, with powerful economic and political elites benefiting from it. Policies that rein in the more extensive interests that trigger the wasteful economy of consumerism entrenched in capitalist societies are required. Hence, there is a need for systematic policy change, with solid political will, and leadership to enable sufficiency, cut on advertising of socially and ecologically damaging sectors, activities ad products. Degrowth policy proposals are developing as we write. For example, Kallis et al. (2020) and Hickel (2021a, b) argue for, among others: carbon and wealth taxes, scaling down environmentally polluting sectors and industries while promoting locally sourced production and consumption economies, universal basic or living income, four days working week, sharing work and in that way reducing inequality, and reintroducing slower lifestyles. An impending EU deforestation regulation aims to ban import of deforestation and forest degradation risk commodities in EU (European Commission 2021). While such efforts are commendable, they do not directly tackle overconsumption or inequality associated with tropical deforestation. Instead, as Zhunusova et al. (2022) argue, they risk becoming a means to shrug a significant part of the responsibility for tackling deforestation onto actors in low-income countries, especially smallholder farmers.

In contrast, the EU deforestation policy should be oriented towards sufficiency, a wellbeing-oriented economy, and reducing overconsumption, rather than substitution with non-renewable energy and materials. Post-growth economic models (Brand et al. 2021; Raworth 2012, 2017) are increasingly recognized as better options to achieve life within planetary boundaries, as well as human well-being (Keyßer and Lenzen 2021). Yet, policy and institutional shifts towards sufficiency and material metabolism are important but insufficient. Broader cultural, epistemic, and equality-oriented goals are needed. A place to start would be to unlearn the EU’s way of doing global environmental politics and development and instead start to listen to and learn from more marginalised groups in the tropical countries, in defining policy problems, designing policy solutions, and implementing them. This by default means rethinking laws and market-oriented policies that aim to “fix the other” and to start self-reflecting and fixing the self.

### Addressing unequal exchange and inequalities through debt cancellation and climate reparations

As noted earlier, SAPReF rightly acknowledges that inequality produces and exacerbates deforestation. This indicates that to be successful, any solution to deforestation must also aspire to address existing inequalities. Confronting material inequality embedded in tropical deforestation requires disentangling who influences production decisions, market prices and the distribution of benefits from agro-commodities trade. In multiple instances, these issues are dominated by transnational corporations, who lobby aggressively to ensure policies main the status quo and the undue advantage it offers them (Bebbington et al. 2018; Blum et al. 2022). The views, interests, and concerns of producer countries and, more importantly, actual producers are usually left unresolved in policies that directly affect them (Partzsch 2017, 2021), as is the case of both policies examined. This translates into the uneven distribution of profits from agro-commodities trade, exploitation, poverty, and even more inequality. The recurring patterns of inequality have evolved over five centuries and cannot be changed with the flip of a switch; instead, conscious, and sustained efforts are required.

Taking responsibility for institutionalizing debt in many countries in the global South as a starting point (“Unequal exchange, power, and economic relations: from modernisation through dependency to degrowth”), it would be essential to explore ways to cancel many of the debts that strangle countries in the South and compel them to exploit their peoples and environment and forest resources, mainly for debt servicing. In addition, climate justice movements (Gonzalez 2020; Sultana 2022) call for reparation for past and present forms of slavery and exploitation can be used as a medium and guidance for addressing inequalities and fostering locally devised and autonomous responses in less wealthy countries. In addition to the above-listed policies, taxing agrocommodities to embody the externalities their production imposes on the environment, and breaking monopolies that transnational companies wield over producers in the global South may slowly bring some form of a balance in the prevailing unequal exchange relations that primarily benefit the global North, economically, while leading to a breakdown in social and environmental systems in the global South. Concurrently, undoing epistemic and ontological injustices that are the foundation of exploitative structures and relations is also needed (Arsenault et al. 2019; Blaser 2010; Trisos et al. 2021), as we will argue next.

### Addressing onto-epistemological inequalities, making space for just and anti-colonial international partnerships

In “Unequal relations and structures of oppression: past, present, and future”, we refer to the multiple philosophies of life, highlighting those rooted in relational ontology, which transcends the binary between human/non-human and fosters reciprocal relations between human and more-than-human beings. Nevertheless, a set of logic, structures and mentalities originating in the colonial project (Grove 1996; Quijano 2007) have continuously marginalised, denied and delegitimized these ontological understandings, rendering them as ‘other’ and ‘traditional’. This resulted in a dominant Eurocentric onto-epistemological knowledge, which was put in use to legitimise extractivist, consumerist and capitalist-based cultures and the imperial mode of living. Knowledge production, to start with, is globally dominated by white scholars (with the privilege of accessing research funds compared to the non-white population) and Eurocentric knowledge systems. This distorts the idea of what counts as ‘scientific’ knowledge, but also of the ways how science is produced and how resources and labour are accessed and achieved—all of which serve the continuity of North–South and racial domination in science, technology, and economy (Collins et al. 2021; Trisos et al. 2021). Thus, anything that lies outside the North’s horizon of knowledge systems and ways of knowing is primarily disregarded as non-scientific, ‘traditional’ and ‘local’, even when such knowledge has proven more effective in achieving better harmony between nature, society and humanity, as in the case of indigenous knowledge systems, for example, Dawson et al. (2021).

The European ‘Will to Improve’ (Li 2007) cannot justify the imposition of ‘well-intended external policies’ on the global South as such policies often create a bureaucratic burden, misrecognition, and slow and epistemic violence (Massarella et al. 2020; Milne and Mahanty 2019; Ramcilovic-Suominen et al. 2021). To serve local notions of justice and anti-colonial agenda, policy actors need to provide more space for customary and indigenous authorities to influence policies rather than relying mainly on state actors and formal structures. Scale is vital; policies should recognise and prioritise Indigenous and otherwise marginalized knowledge systems, but also local political agency, together with the right to self-determination and self-governance (Arsenault et al. 2019; Ndlovu-Gatsheni 2020).

A more just and anti-colonial agenda requires recognising that the colonial project was instrumental in establishing Europe as a dominant power and wealthy region (Hickel 2018). Second, it requires public and intellectual engagement with the idea that the colonial and postcolonial structures continue to shape the political and economic relations (Latouche 2014; Mignolo and Walsh 2018). More just international relations require repositioning the EU and other global hegemonic actors in the world (Delputte and Orbie 2020), followed by a fresh approach to partnerships based on anti-colonial and decolonial values and praxis (Mignolo and Walsh 2018; Tuck and Yang 2012). For SAPReF and the UBS, responding to such calls means shifting from the Eurocentric framings of tropical deforestation as the governance problem of the less wealthy countries to recognising the North’s own historical debts and current shortcomings.

Generally, anti-colonial and decolonial agendas and concerns are yet to adequately penetrate EU policy discourses and policy-making processes. It is understandable that many of the claims of such an agenda unsettle the established system and logic of competitiveness, dominance and reproducing existing dependency relations—yet they are necessary to safeguard life in the race to extinguish the extinction of existences caused by current exploitative systems in place in the global South (Ramcilovic-Suominen et al. 2021; Rutazibwa 2018). Therefore, we contend that the ideas from decolonial thinking need to become more integral to the EU bioeconomy and larger development trajectories. Such ideas hold the potential to transform the EU by enabling it to establish fairer ways of relating with its former colonies (Rutazibwa 2018) and overcoming how it currently exploits and consumes resources from them (Eversberg 2020; Grove 1996). Shifting rhetoric and terminology from “Commission for Development” to “International partnerships” is insufficient if practices remain essentially unchanged. The shift in rhetoric needs to be met with equivalent concrete actions and measures where the EU would act on, among others, reparations, and climate taxes, in the spirit of climate justice and out of recognition and respect for other ways of being.

## Concluding remarks

The remaining forests in multiple tropical countries are under severe pressure due to agro-commodities and biomass production for European and other advanced economies. While the EU is not the only actor responsible for tropical deforestation, it is a significant player, and the EU policies recognise this. However, the lack of adequate and concrete measures to reduce the pressure on deforestation-risky commodities, and reduce the politics of domination, poses the question of whether the EU is avoiding responsibility for tropical deforestation while claiming an ever-increasing right to manage and use lands, labour, and resources from the global South. The question is especially relevant in the light of the EU’s agenda to advance a bio-based economy and its green transition, aiming to shift from fossil to biobased materials and renewable energy infrastructure. The EU appears to be taking various initiatives, from voluntary schemes to due diligence market-based proposals, to tackle tropical deforestation and the socio-ecological crises it creates. Many of the plans advanced under the EU’s SAPReF and the UBS’ have a place in addressing tropical deforestation, but not in a manner that would lead to significant or more just results. Conversely, they massage rather than confront the deeply rooted, structural causes of tropical deforestation, including the inequality embedded in and reproduced by agro-commodities production, consumption, and trade. Without confronting these issues, any new policy proposals, like previous attempts (Heflich 2020), may not succeed in halting tropical deforestation.

To achieve meaningful progress in tackling deforestation, the EU needs to confront its internal appetite and proclivity to overconsume deforestation-causing commodities, using the existing (neo)colonial mechanisms and structures of oppression, and instead promote policies that serve and enable more ecologically sustainable, self-sufficient, and just transitions to European biosociety. Such policies need to advance anti-imperialist, anti-colonial and post-growth agendas, which call for repairing and addressing past and present inequalities associated with production and trade of tropical agro-commodities. Cancelling the debts of previously colonised countries that are currently less wealthy, taxing agro-commodities companies to internalise the effects their sourcing has on the environment and people, and adequately regulating and monitoring transnational corporations to reduce their exploitation of producers in deforestation-risk countries are options to consider. Furthermore, anti-colonial policies and praxis that strengthen locally defined and designed approaches that support local autonomy, local knowledge systems, sovereignty, and independence are articulated.

It is worth repeating that deforestation is only a symptom of deep-rooted inequities in the global economy. Addressing these root causes requires a shift in logic and motivations, including global domination, competitiveness and pursuing economic growth at all costs. Hence, if the EU wants to live up to Angela Merkel’s plea, during the 2021 One Planet Summit, “*to protect biodiversity and natural habitats—not some time or other, but now, and not somehow or other, but monumentally*” (Merkel 2021, p. 1), it must start by questioning its own values and logics, together with its dream for green capitalist economic system.

## Data Availability

Data sharing not applicable—no new data generated.

## Footnotes

1 The IRP uses material footprint to characterize ‘all of the material resources mobilized globally to the final consumer, and so it traces embodied or virtual flows of materials associated with value, rather than simply territorially delineated physical flows’ (IRP 2019, p. 51).

2 Inequalities are also significant between and within countries in the North and South (see Chancel 2022).
